# *NME5* frameshift variant in Alaskan Malamutes with primary ciliary dyskinesia

**DOI:** 10.1371/journal.pgen.1008378

**Published:** 2019-09-03

**Authors:** Linda Anderegg, Michelle Im Hof Gut, Udo Hetzel, Elizabeth W. Howerth, Fabienne Leuthard, Kaisa Kyöstilä, Hannes Lohi, Louise Pettitt, Cathryn Mellersh, Katie M. Minor, James R. Mickelson, Kevin Batcher, Danika Bannasch, Vidhya Jagannathan, Tosso Leeb

**Affiliations:** 1 Institute of Genetics, Vetsuisse Faculty, University of Bern,Bern, Switzerland; 2 Kleintierpraxis Laupeneck, Bern, Switzerland; 3 Institute of Veterinary Pathology, Vetsuisse Faculty, University of Zurich, Zurich, Switzerland; 4 Department of Pathology, College of Veterinary Medicine, University of Georgia, Athens GA, United States of America; 5 Department of Veterinary Biosciences, University of Helsinki, Helsinki, Finland; 6 Department of Medical and Clinical Genetics, University of Helsinki, Helsinki, Finland; 7 Folkhälsan Research Center, Helsinki, Finland; 8 Kennel Club Genetics Centre, Animal Health Trust, Newmarket, Suffolk CB UU, United Kingdom; 9 Department of Veterinary and Biomedical Sciences, University of Minnesota, Saint Paul, MN, United States of America; 10 Department of Population Health and Reproduction, University of California-Davis, Davis, CA, United States of America; Stanford University School of Medicine, UNITED STATES

## Abstract

Primary ciliary dyskinesia (PCD) is a hereditary defect of motile cilia in humans and several domestic animal species. Typical clinical findings are chronic recurrent infections of the respiratory tract and fertility problems. We analyzed an Alaskan Malamute family, in which two out of six puppies were affected by PCD. The parents were unaffected suggesting autosomal recessive inheritance. Linkage and homozygosity mapping defined critical intervals comprising ~118 Mb. Whole genome sequencing of one case and comparison to 601 control genomes identified a disease associated frameshift variant, c.43delA, in the *NME5* gene encoding a sparsely characterized protein associated with ciliary function. *Nme5*^*-/-*^ knockout mice exhibit doming of the skull, hydrocephalus and sperm flagellar defects. The genotypes at *NME5*:c.43delA showed the expected co-segregation with the phenotype in the Alaskan Malamute family. An additional unrelated Alaskan Malamute with PCD and hydrocephalus that became available later in the study was also homozygous mutant at the *NME5*:c.43delA variant. The mutant allele was not present in more than 1000 control dogs from different breeds. Immunohistochemistry demonstrated absence of the NME5 protein from nasal epithelia of an affected dog. We therefore propose *NME5*:c.43delA as the most likely candidate causative variant for PCD in Alaskan Malamutes. These findings enable genetic testing to avoid the unintentional breeding of affected dogs in the future. Furthermore, the results of this study identify *NME5* as a novel candidate gene for unsolved human PCD and/or hydrocephalus cases.

## Author summary

Motile cilia are required for clearing mucous, infectious agents and inhaled dust from the airways. Primary ciliary dyskinesia (PCD) is a hereditary defect of motile cilia. Clinical findings may include recurrent airway infections, fertility problems, and sometimes hydrocephalus. We analyzed an Alaskan Malamute family, in which two out of six puppies were affected by an autosomal recessive form of PCD. Whole genome sequencing of an affected dog identified a one base pair deletion in the *NME5* gene, c.43delA, leading to an early frame-shift and premature stop codon. Later in the study, we became aware of a previously published Alaskan Malamute with PCD involving respiratory infections and hydrocephalus. We observed perfect concordance of the *NME5* genotypes with the PCD phenotype in all three affected Alaskan Malamutes and more than 1000 controls. The fact that the third case, which had no documented close relationship to the initial two cases, was homozygous for the same rare mutant *NME5* allele, strongly supports our hypothesis that *NME5*:c.43delA causes the PCD phenotype. We confirmed absence of *NME5* protein expression in nasal epithelium of an affected dog. Our results enable genetic testing in dogs and identify *NME5* as novel candidate gene for unsolved human PCD cases.

## Introduction

Primary ciliary dyskinesia (PCD) is a rare genetic disease caused by defects in the structure or function of the motile cilia. Motile cilia are present in the respiratory tract including the paranasal sinuses, in the auditory tube and middle ear, in male and female reproductive tracts, sperm cells, and in the ependyma lining the ventricular system and central canal of the brain and spinal cord. Abnormal ciliary function typically leads to recurrent and chronic infections of the upper and lower respiratory tract beginning in neonates due to reduced mucociliary clearance [1].

Bronchiectasis, recurrent ear infections and infertility are also common findings in patients with PCD. During embryogenesis, cilia are important to establish correct left-right asymmetry. Therefore, *situs inversus* is present in approximately 50% of PCD patients [2,3]. Primary ciliary dyskinesia with *situs inversus* has been termed Kartagener syndrome [4].

Motile cilia have a characteristic 9 + 2 structure with nine microtubular doublets arranged in a circle around a central pair of microtubules. Additional ultrastructural elements, such as the outer and inner dynein arms or the radial spokes are important for the proper function of motile cilia [3,5].

Most forms of PCD are inherited with an autosomal recessive mode of inheritance. However, cases with autosomal dominant or X-linked mode of inheritance have also been described [6,7]. In humans, variants in 40 different genes have been reported to cause PCD [3,8,9].

PCD is also known in dogs and has been described in numerous dog breeds including Alaskan Malamutes and mongrels [10–23]. The genetic analysis of PCD affected Old English Sheepdogs unveiled a missense variant in *CCDC39* as causative variant and led to the subsequent discovery of *CCDC39* variants in human PCD patients [11,12]. To the best of our knowledge, no other canine PCD causative variant has been reported in the literature.

In this study we describe the clinical, pathological and genetic analysis of PCD in Alaskan Malamutes.

## Results

### Family history and clinical findings

An Alaskan Malamute litter with six puppies originating in Switzerland initiated the study. A few days after birth, two intact female puppies presented with bilateral mucoid to mucopurulent nasal discharge and subsequent chronic productive cough. Neither the puppies’ siblings nor the parents showed similar clinical signs. The affected puppies were in good body condition, bright, alert and responsive. Increased lung sounds were identified on thoracic auscultation in both dogs.

### Further investigations

**Fig 1:**
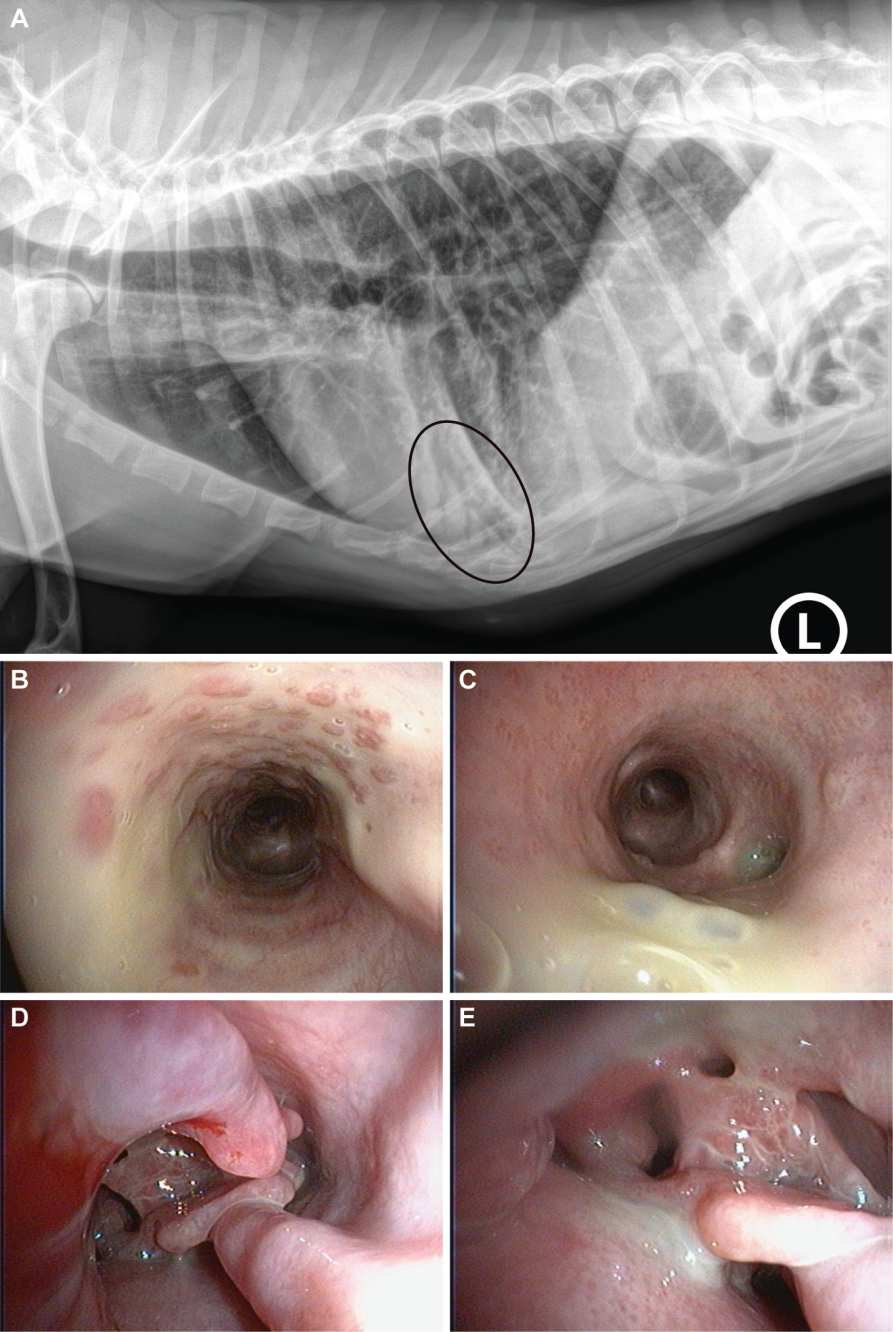
PCD phenotype in Alaskan Malamutes. **(A)** Left lateral view of the thorax of one of the affected dogs at the age of 15 months with bronchopneumonia in the right middle lung lobe (encircled). **(B)** Endoscopic image of the trachea. Mucosal hyperemia with cobblestone appearance covered with a large amount of mucopurulent secretion. **(C)** Endoscopic image of the right caudal lung. Hyperemic bronchi with large amount of mucopurulent secretions is evident. **(D, E)** Endoscopic images of the nasal cavity. Severe turbinate lysis, hyperemic mucosa and mucopurulent mucus is evident. The bleeding on the left is from the endoscope and represents an iatrogenic lesion.

Hematology and biochemistry was normal in one dog and revealed abnormalities consistent with chronic non-specific inflammation in the other dog. Further investigations revealed severe bronchial lung pattern and bronchiectasis on thoracic radiographs in both dogs and abnormalities compatible with bronchopneumonia in one dog (Fig 1). No evidence of *situs inversus* was seen in the radiographs of the affected dogs.

Direct rhinoscopy and bronchoscopy revealed hyperemic mucosa, medium to large amount of mucopurulent secretions along the upper and lower airway tracts and moderate to severe turbinate lysis in the nasal cavity in both dogs (Fig 1).

Bronchoalveolar lavage fluid was compatible with chronic active purulent bronchopneumonia. Microbial culture yielded β-haemolytic *Streptococcus* in one dog and *Pseudomonas fluorescens* and *Pasteurella multocida* in the other dog. Parasites or fungi were not detected.

### Histological findings and transmission electron microscopy

**Fig 2:**
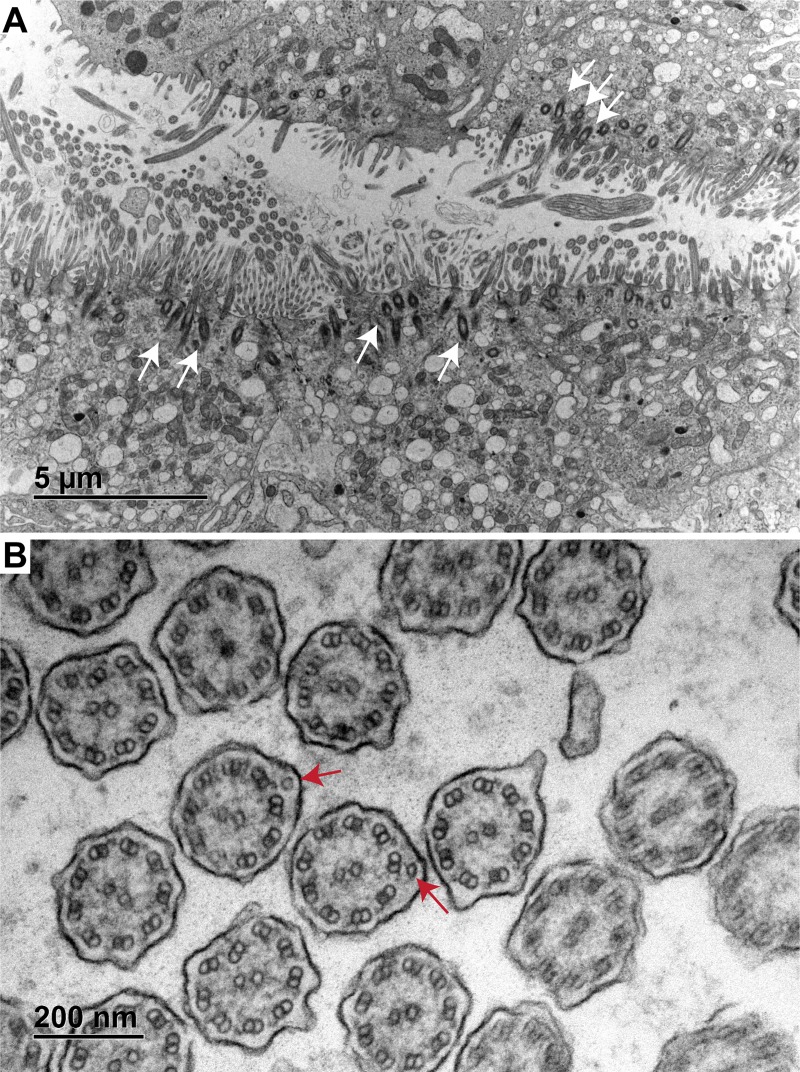
Transmission electron micrographs of bronchial mucosa from a PCD affected Alaskan Malamute. **(A)** Overview of a ciliated airway demonstrating an overall reduced ciliation (upper part) with prominent basal bodies (white arrows). **(B)** A cross section of cilia is shown at higher magnification. In normal cilia, there is a 9 + 2 arrangement of microtubules with two single microtubules in the center and nine pairs of peripheral microtubules. In the affected dog, extra peripheral microtubule singlets appeared occasionally (red arrows). Furthermore, some of the outer dynein arms and most of the inner dynein arms were shortened or entirely absent.

Biopsies of nasal and bronchial mucosa from the two affected dogs were examined. In the first puppy, the nasal mucosa had a reduced number of cilia and showed moderate purulent rhinitis. The bronchial mucosa also showed signs of mild chronic purulent bronchitis. Ultrastructural examination of the cilia from bronchial mucosa revealed a small proportion of cilia with an abnormal 10 + 2 conformation of the microtubules. Furthermore, about 60% of the outer and 95% of the inner dynein arms were shortened or absent (Fig 2).

Nasal and bronchial samples of the second puppy revealed similar, but more pronounced ciliary alterations. Inner dynein arms were absent in nearly 100% of cilia, outer arms were either extremely shortened or absent in about 80% of the cilia. In addition, compound cilia, absence of one or both central complex tubules, reduction of microtubular singlet or doublets and disarrangement of tubules were observed.

### Genetic mapping of the disease-causing variant

**Fig 3:**
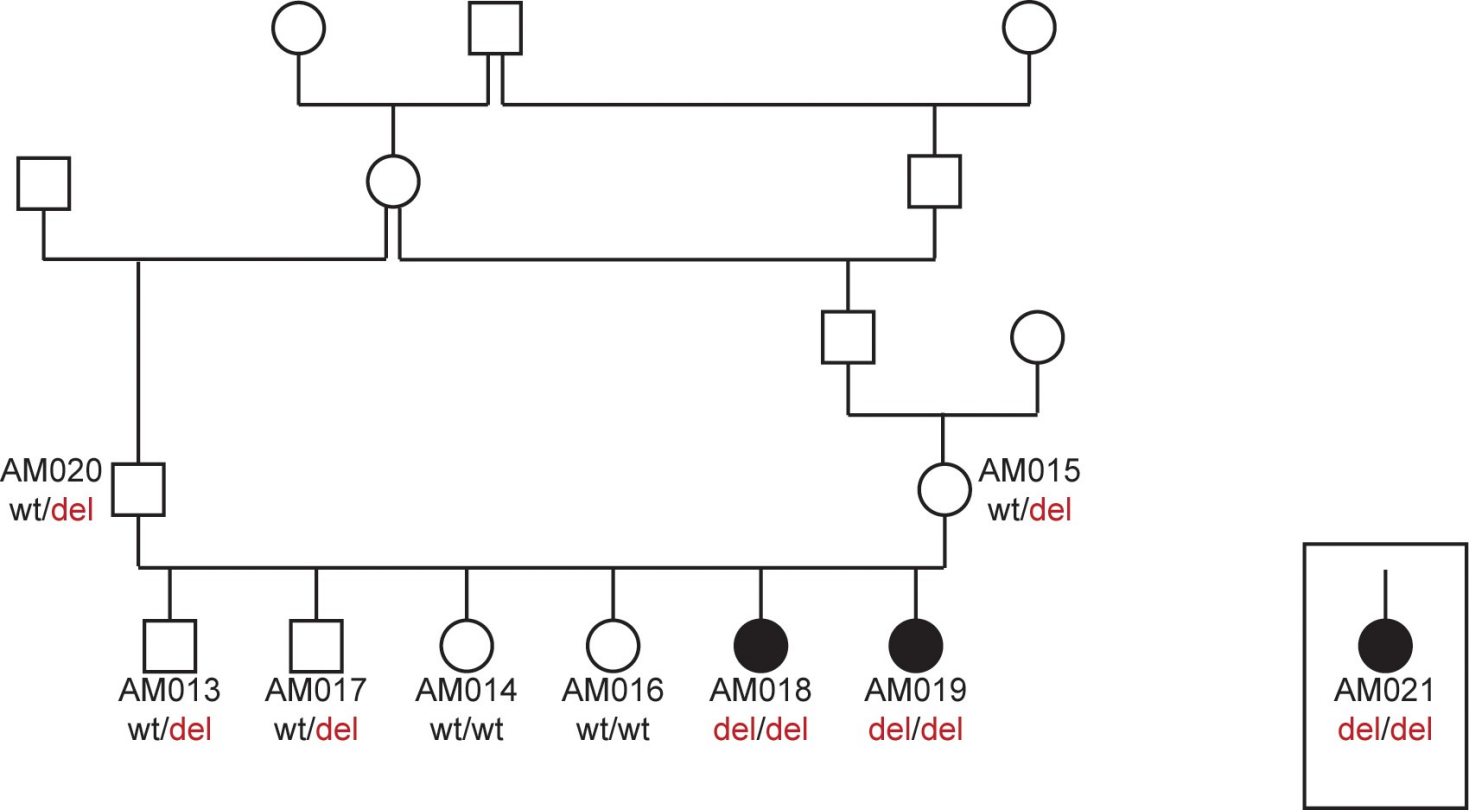
Pedigree of the Alaskan Malamute family with two PCD cases. Filled symbols represent dogs affected by PCD. The solitary symbol in the square represent the additional PCD affected dog (AM021) previously reported in the USA of which no pedigree data was available. For genotyping, DNA of this dog was extracted from FFPE tissues [10]. Other lab numbers indicate dogs, of which blood samples were available. Genotypes of the *NME5*:c.43delA variant for these dogs are shown. Two Inbreeding loops are visible in this pedigree.

**Fig 4:**
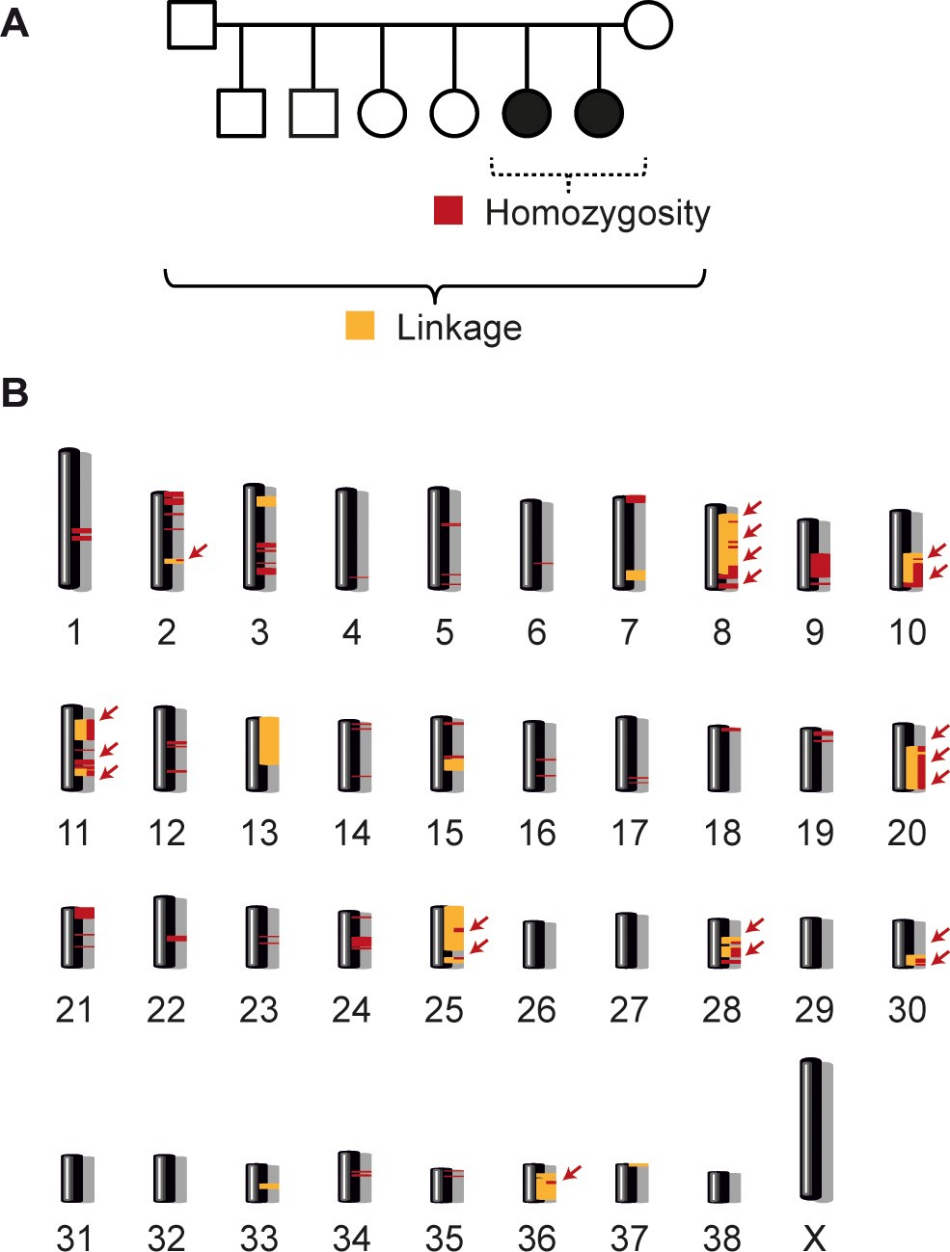
Combined linkage analysis with homozygosity mapping. **(A)** Parametric linkage analysis was performed with eight family members of one Alaskan Malamute family. Homozygosity mapping was made with two affected dogs from this family. **(B)** Linked regions >1 Mb are marked in yellow and all shared homozygous regions are marked in red. Twenty regions on different chromosomes showed overlapping linked and homozygous regions (arrows) which are designated as critical intervals.

The pedigree of the two affected puppies with a documented inbreeding loop suggested an autosomal recessive mode of inheritance (Fig 3). Linkage analysis in the available family identified 20 linked genome segments totaling 319 Mb. We additionally performed homozygosity/autozygosity mapping in the two affected littermates. They shared 63 homozygous segments >1 Mb with identical alleles. The intersection of the linked and homozygous intervals comprised 20 chromosome segments spanning 117,799,906 bp (Fig 4; S1 Table).

### Identification of a candidate causative variant

We sequenced the genome of one PCD affected Alaskan Malamute at 36x coverage and called single nucleotide variants (SNVs) and small indel variants with respect to the CanFam 3.1 reference genome. We then compared these variants to whole genome sequence data from 8 wolves and 593 control dogs from genetically diverse breeds (Table 1, S2 Table). We identified seven private homozygous protein-changing variants in the critical intervals of the case genome (Table 2).

**Table 1:** Variants detected by whole genome resequencing of a PCD affected dog.

Filtering step	Number of variants
Homozygous variants in the whole genome	3,287,471
Private homozygous variants in the whole genome^a^	8,877
Private homozygous protein changing variants in the whole genome^b^	45
Private homozygous protein changing variants in critical interval	7

^a^ Private variants were exclusively present in the affected dog and absent from 601 control genomes.

^b^ Variants with SnpEff impact predictions “high” and “moderate”.

**Table 2:** Private homozygous protein changing variants in the PCD affected dog.

Chr.	Position	Gene	Transcript	Variant cDNA	Variant protein
11	25,792,084	*NME5*	XM_003639378.4	c.43delA	p.Thr15LeufsTer56
20	48,455,036	*PALM3*	XM_014121803.2	c.634T>A	p.Ser212Thr
20	49,352,482	*PRDX2*	XM_022406985.1	c.67C>T	p.Arg23Trp
20	49,537,837	*LOC484934*	XM_542050.6	c.883C>G	p.Arg295Gly
20	56,148,068	*TLE2*	XM_022407255.1	c.698A>G	p.Lys233Arg
20	56,474,502	*DIRAS1*	XM_005633100.3	c.398G>A	p.Arg133His
20	56,784,636	*JSRP1*	XM_022407394.1	c.629C>T	p.Ala210Val

At this stage of the project, we became aware of another previously reported PCD affected Alaskan Malamute from the United States. This dog had persistent nasal discharge starting before the age of six weeks. Due to chronic respiratory infections, the dog was euthanized at 8 months of age. At necropsy, PCD with bronchopneumonia, bronchiectasis and hydrocephalus were diagnosed. Abnormal cilia arrangement lacking inner and outer dynein arms was found in transmission electron microscopy [10].

**Fig 5:**
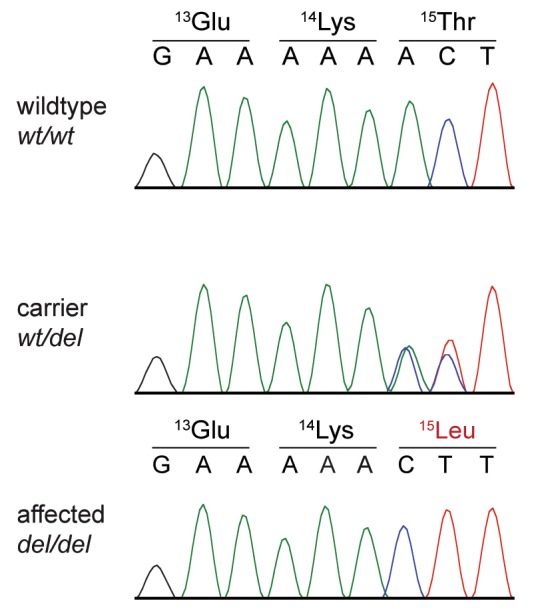
Sanger sequencing of the *NME5*:c.43delA variant. Electropherograms from dogs with the three different genotypes confirm the presence of the variant.

We genotyped the seven private protein-changing variants on DNA from an archived FFPE tissue sample in the additional American PCD case. The additional case was homozygous for the alternative allele at only one of the seven variants, a frame-shifting single base deletion in the second exon of the *NME5* gene (S3 Table). The full designation of this variant is XM_003639378.4:c.43delA. It is predicted to result in an early premature stop codon, which truncates more than 90% of the wildtype NME5 protein, XP_003639426.1:p.(Thr15LeufsTer56). The variant and the expected co-segregation with the PCD phenotype in the family were confirmed by Sanger sequencing (Fig 5; S3 Table).

We further genotyped 404 Alaskan Malamute samples and 539 control dogs from 72 genetically diverse breeds. This experiment confirmed the perfect association between the genotypes at *NME5*:c.43delA and the PCD phenotype. The mutant allele was not detected outside of the Alaskan Malamute breed (Table 3, S3 Table).

**Table 3:** Association of the *NME5*:c.43delA genotype with the PCD phenotype.

*NME5*:c.43delA genotype	wt/wt	wt/del	del/del
Alaskan Malamute cases (n = 3)	-	-	3
Alaskan Malamute obligate carriers (n = 2)^a^	-	2	-
Alaskan Malamute controls (n = 402)	397	5^b^	-
Dogs from other breeds (n = 539)^c^	539	-	-

^a^Parents of the two Swiss PCD cases.

^b^Two of the heterozygous dogs (presumed carriers) were littermates of the two Swiss PCD cases.

^c^These 539 dogs were independent from the 593 dogs and 8 wolves with WGS data. Thus, the variant was absent from > 1000 dogs and wolves outside of the Alaskan Malamute breed.

### Functional confirmation at the protein level

**Fig 6:**
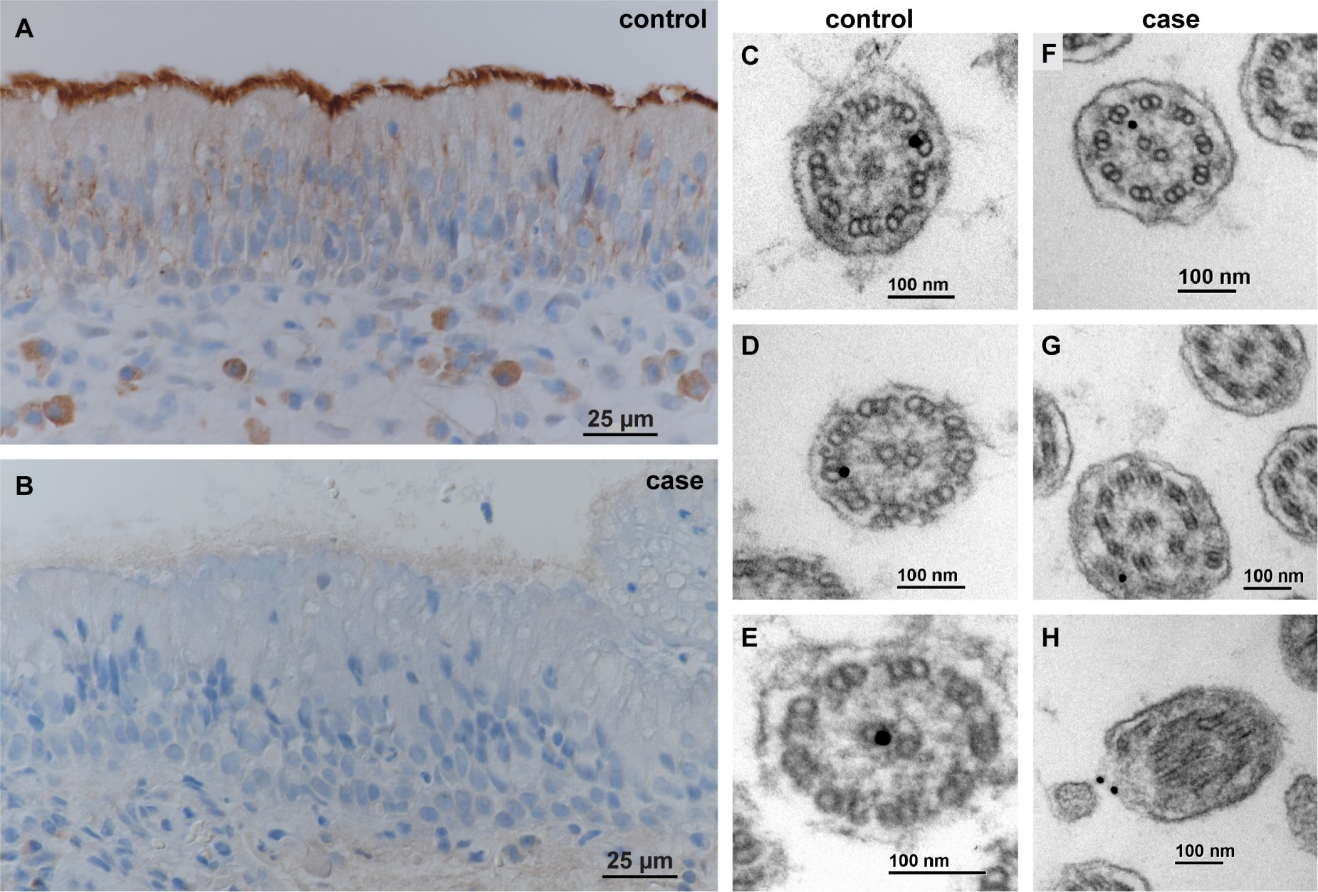
NME5 protein expression. (**A**) NME5 immunohistochemistry shows a strong signal at the ciliated epithelium from nasal mucosa of a control dog. (**B**) In a PCD affected Alaskan Malamute, the same polyclonal anti-NME5 antibody does not give any detectable reaction. (**C-H**) NME5 immunogold transmission electron microscopy of nasal mucosa, ciliary cross sections. (**C-E**) Nasal mucosa of a control dog with positive binding of gold particles to (**C,D**) outer microtubules at the inner dynein arm location and to (**E**) the central microtubules. (**F-H**) Nasal mucosa of a PCD affected Alaskan Malamute with single positive gold particles binding to different locations within the cilia, possibly due to non-specific binding of the antibody.

We investigated the NME5 protein expression in nasal mucosa by immunohistochemistry. While there was a strong positive signal in the nasal epithelium from an unaffected control, NME5 protein expression was not detectable in the nasal mucosa of a PCD affected Alaskan Malamute (Fig 6A and 6B).

NME5 immunogold transmission electron microscopy on nasal mucosa specimens demonstrated an overall low positive antibody binding in cilia from the control dog (5/20 cilia). Outer and inner microtubuli showed positive reactions. The most frequently observed binding sites were in the region of the inner dynein arms in the proximity of the outer microtubuli (Fig 6C, 6D and 6E). In nasal ciliated epithelium from the affected dog, there were less positive signals than in the control (3/20 cilia). The antibody binding sites appeared more irregular and were different from the binding localizations of the control specimen (Fig 6F, 6G and 6H).

## Discussion

The present investigation identified the *NME5*:c.43delA frameshift variant as most likely candidate causative genetic variant for an autosomal recessive form of PCD in Alaskan Malamutes. The perfect genetic association in a large cohort of dogs, correct segregation of the mutant allele in the Swiss family, and demonstrated absence of NME5 protein expression in nasal epithelia from an affected dog all support the causality of *NME5*:c.43delA.

The *NME5* gene encodes the NME/NM23 family member 5, also known as nonmetastatic protein 23, homolog 5 (NM23H5) or radial spoke 23 homolog (*Chlamydomonas*; RSPH23). The *NME* gene family contains 10 paralogs in humans (*NME1* –*NME10*) [24]. The encoded proteins share a conserved 152 amino acid nucleoside diphosphate kinase domain. RSP23, the *Chlamydomonas* ortholog of NME5, has been shown to associate with the radial spoke necks of flagellar cilia. RSP23 has a nucleoside kinase activity and was hypothesized to be involved in GTP generation required for flagellar beating in *Chlamydomonas* [25, 26]. However, the vertebrate NME5 has lost its nucleoside kinase activity, and its precise function remains elusive [27]. Human NME5 has been detected in sperm flagella [27,28] and more recently also been identified as a lowly abundant component of the radial spokes in human airway cilia [29].

Our data confirm that NME5 is expressed in ciliated airway epithelia and suggest that it is a lowly abundant component of cilia, which is tightly associated with the central or peripheral microtubules. This localization is consistent with previously reported findings in human sperm flagella [27]. Our ultrastructural localization must be interpreted with caution as it is based on a routine diagnostic specimen from a single unaffected dog. Nonetheless, a physiological localization at the peripheral microtubule pairs fits well with the observed defects in the outer and particularly inner dynein arms present in NME5 mutant dogs with PCD.

To the best of our knowledge, no human patients with genetic variants in *NME5* have been reported. *Nme5*^-/-^ knockout mice have functional defects in their motile cilia. Their phenotype is primarily characterized by doming of the skull together with moderate to marked hydrocephalus. In male *Nme5*^*-/-*^ knockout mice, late-stage spermatogenic arrest and flagellar defects were noticed. The cilia on respiratory epithelium and ependymal cells had a histologically normal appearance. However, it was also described that several of the *Nme5*^-/-^ knockout mice had proteinaceous and suppurative exudates in nasal sinuses and passageways [30].

The two Swiss and the American case showed comparable clinical signs including bronchopneumonia and bronchiectasis. Post mortem necropsy in the American case revealed a hydrocephalus in addition to the changes seen in the airways [10]. It remains unclear whether the two Swiss cases have evidence of hydrocephalus, as they were still alive when the study was completed. The Swiss cases did not show any neurological signs and were thus not subjected to an MRI to evaluate the presence of hydrocephalus.

The ultrastructural changes in the cilia, which were most prominent for the inner and outer dynein arms, were comparable in the affected Alaskan Malamutes from Switzerland and America [10]. All the phenotypic parallels between the three reported cases suggest a common underlying etiology.

Recurrent respiratory infections represented the primary clinical sign in the affected dogs. This is quite distinct from the *Nme5*^-/-^ knockout mice, in which hydrocephalus was the predominant feature. However, both entities are the result of motile cilia dysfunction. Hydrocephalus may be caused by deficient motility of ependymal cilia during brain morphogenesis and a subsequent altered flow of cerebrospinal fluid between the ventricles [30]. The additional Alaskan Malamute case with PCD and hydrocephalus from the United States illustrates the phenotypic similarities between *NME5* mutant mice and dogs. Mice might be particularly sensitive to develop hydrocephalus as consequence as their cerebral aqueduct is relatively long and narrow [30]. None of the *Nme5*^-/-^ knockout mice or *NME5* mutant dogs had *situs inversus totalis* indicating that NME5 may be dispensable for the establishment of a correct left/right asymmetry.

In conclusion, our data identify *NME5*:c.43delA as most likely causative variant for PCD in Alaskan Malamutes. This form of PCD can be associated with hydrocephalus. Our findings enable genetic testing to avoid unintentional breeding of affected dogs in the future. Furthermore, *NME5* should be considered as candidate gene for unsolved human PCD and/or hydrocephalus cases.

## Materials and methods

### Ethics statement

All animal experiments were performed according to the local regulations. The dogs in this study were examined with the consent of their owners. The study was approved by the “Cantonal Committee For Animal Experiments” (Canton of Bern; permit 75/16).

### Animals and samples

This study included samples from 407 Alaskan Malamutes (3 PCD cases / 404 controls). Two cases were female littermates originating from Switzerland. The third, previously reported case originated from the USA and was only included after the completion of the whole genome sequencing experiments [10]. The three PCD cases were diagnosed due to abnormal findings of motile cilia structure in transmission electron microscopy. The remaining 404 Alaskan Malamutes represented population controls for which we had no reports of PCD. These samples came from different collections: 19 from the Vetsuisse Biobank (Switzerland), 153 from Finland, 120 from the United Kingdom and 112 from the United States. As additional controls, we used samples of 539 dogs from 72 different other breeds, which had been donated to the Vetsuisse Biobank (S4 Table).

### Physical examination and further investigations

Two affected Swiss female littermates were referred to a diplomate of the American College of Veterinary Internal Medicine (MIHG) for diagnostic investigations of chronic nasal discharge at the age of 5 months and 18 months respectively. Physical examination and thoracic radiographs were performed in both affected dogs. Abdominal radiographs were performed in one dog.

Due to the neonatal onset of the disease and its chronic recurrent course of the two littermates, primary ciliary dyskinesia was suspected. Blood samples for hematology, biochemistry and DNA extraction were obtained.

Rhinoscopy and bronchoscopy were performed under general anesthesia and allowed macroscopic evaluation and biopsy sampling of the upper and lower respiratory airways. Bronchoalveolar lavages were cultured for bacteria and examined cytologically. Nasal and bronchial biopsies were fixed in formalin and 2.5% glutaraldehyde.

### Transmission electron microscopy

Tissue samples for transmission electron tissue were fixed with 2.5% glutaraldehyde (EMS) in 0.1 M sodium phosphate buffer (pH 7.4) overnight and washed three times in 0.1 M sodium phosphate buffer. Specimens were post fixed in 1% osmium tetroxide (Sigma-Aldrich) and dehydrated in an ascending ethanol series followed by propylen oxide and infiltration in 30% and 50% Epon (Sigma-Aldrich). At least three 0,9 μm toluidine blue stained semithin sections per localisation were produced. Representative areas were trimmed. Subsequently, 90 nm lead citrate (Merck) and uranyl acetate (Merck) contrasted ultrathin sections were produced and viewed under a Phillips CM10 transmission electron microscope, operating with Gatan Orius Sc1000 (832) digital camera and Gatan Microscopical Suite, Digital Micrograph, Version 230.540.

### DNA isolation

Genomic DNA was extracted from EDTA blood samples according standard methods using the Maxwell RSC Whole Blood DNA kit in combination with the Maxwell RSC machine (Promega). Genomic DNA from formalin-fixed paraffin-embedded (FFPE) tissue samples was extracted using the Maxwell RSC DNA FFPE kit according manufacturer’s instructions.

### SNV genotyping

Eight dogs were genotyped for 220,853 SNVs on the illumina canine_HD chip by GeneSeek/Neogen. The raw SNV genotypes are given in the S1 File.

### Linkage analysis and homozygosity mapping

We performed linkage analysis and homozygosity mapping with eight dogs from one family (Figure Mapping). The genotype data of these eight dogs from Illumina canine_HD chip were used for a parametric linkage analysis. For all dogs, the call rate was > 95%. Using PLINK v 1.09 [31], markers that were non-informative, located on the sex chromosomes, or missing in any of the eight dogs, had Mendel errors, or a minor allele frequency < 0.05, were removed. The final pruned dataset contained 81,116 markers. For parametric linkage analysis, an autosomal recessive inheritance model with full penetrance, a disease allele frequency of 0.4 and the Merlin software [32] were applied.

For homozygosity mapping, the genotype data of two affected dogs from this litter were used. Markers that were missing in one of the two cases, markers on the sex chromosomes and markers with Mendel errors in the family were excluded. Regions of homozygosity with shared alleles between the cases were identified by using the—homozyg and—homozyg group options in PLINK.

### Whole genome sequencing of an affected Alaskan Malamute

An Illumina PCR-free TruSeq fragment library with 400 bp insert size of a PCD affected Alaskan Malamute (AM019) was prepared. We collected 286 million 2 x 150 bp paired-end reads on a NovaSeq 6000 instrument (36x coverage). Mapping and alignment were performed as described [33]. The sequence data were deposited under the study accession PRJEB16012 and sample accession SAMEA4848707 at the European Nucleotide Archive.

### Variant calling

Variant filtering was performed as previous described [33]. SnpEFF software [34] was used to predict the functional effects of the called variants together with the NCBI annotation release 105 on the CanFam 3.1 reference assembly. To identify case-specific private variants we used 601 control genomes, which were either publicly available [35] or produced during other projects of our group or contributed by members of the Dog Biomedical Variant Database Consortium. The accession numbers of all used genomes are given in S5 Table.

### Gene analysis

We used the dog CanFam 3.1 reference genome assembly for all analyses. Numbering within the canine *NME5* gene corresponds to the NCBI RefSeq accessions XM_003639378.4 (mRNA) and XP_003639426.1 (protein).

### Sanger sequencing

We used Sanger sequencing to confirm the *NME5*: c.43delA variant and to genotype the other dogs included in this study. A 312 bp PCR product was amplified from genomic DNA using the AmpliTaqGold360Mastermix (Life Technologies) together with primers 5‘-TCG AAA AAG ATT CGG CAG TT -3‘ (Primer F) and 5’- TCA TCA TGC CCA GAA GTT ACC -3’ (Primer R). For the FFPE sample, a 171 bp PCR product was amplified from genomic DNA using the AmpliTaqGold360Mastermix (Life Technologies) together with primers 5’- TAC CCT GGA AAG GCA GAA TG -3’ (Primer FFPE F) and 5’- CAT CAT CAT CAT GCC CAG AA -3’ (Primer FFPE R). After treatment with exonuclease I and alkaline phosphatase, amplicons were sequenced on an ABI 3730 DNA Analyzer (Life Technologies). Sanger sequences were analyzed using the Sequencher 5.1 software (GeneCodes).

### Immunohistochemistry

For immunohistochemistry investigations, nasal mucosa samples were fixed in 3.5% buffered formaldehyde and routinely processed for paraffin embedding, 3 μm sections were produced and after antigen retrieval with citrate buffer (1h, room temperature, pH 6.0), sections were incubated with polyclonal anti-rabbit Anti-NME5 antibody (Abcam ab231631, dilution 1:800), followed by horseradish peroxidase-labeled goat anti-rabbit antibody (UltraVision anti-rabbit HRP detection system, Thermo Scientific), with subsequent visualization with diaminobenzidintetrahydrochloride (DAB). As non-altered specimen (control), a sample from a normal nasal mucosa of a 1.5 year old, male castrated mixed breed dog was used.

### Immunogold transmission electron microscopy

For immunogold transmission electron microscopy, anti-NME5 polyclonal antibody was applied for 4 h at room temperature in 1:10 dilution after pretreatment of grids in acidic citrate buffer for 16 h at 60°C. The secondary antibody (18 nm Gold Goat Anti-Rabbit IgG; Jackson Immuno Research, 111-215-144) was applied at room temperature for 2 h, dilution 1:20, in Dako Antibody Diluent (Dako REAL). Subsequently, 90 nm ultrathin specimens were routinely contrasted with lead citrate (Merck) and uranyl acetate (Merck).

## Supporting information

S1 File SNV genotype data from Alaskan Malamute family. (ZIP)

S1 Table Data from linkage and homozygosity analyses. (XLSX)

S2 Table Private variants. (XLSX)

S3 Table Genotypes for seven protein changing variants in 22 Alaskan Malamutes. (XLSX)

S4 Table Control dogs genotyped for the *NME5*:c.43delA variant. (XLSX)

S5 Table Public genome accessions. (XLSX)
